# Direct chemical lithography writing on 2D materials by electron beam induced chemical reactions†

**DOI:** 10.1039/d5na00036j

**Published:** 2025-02-10

**Authors:** Iryna Danylo, Lukáš Koláčný, Kristína Kissíková, Tomáš Hartman, Martina Pitínová, Jiří Šturala, Zdeněk Sofer, Martin Veselý

**Affiliations:** a Department of Organic Technology, University of Chemistry and Technology Prague Czech Republic veselyr@vscht.cz; b Department of Inorganic Chemistry, University of Chemistry and Technology Prague Czech Republic soferz@vscht.cz

## Abstract

Due to their high surface-to-volume ratio and native band gaps, two-dimensional (2D) materials are widely used as supports for metal nanoparticle (NP) catalysts. Various synthesis methods exist to prepare such materials, but controlling the amount, size, and distribution of the deposited NPs remains a challenge. Here, we investigate the use of electron beam lithography (EBL) for this purpose. A dual-beam focused ion beam-scanning electron microscope (FIB-SEM) was used to direct the deposition of platinum NPs (Pt NPs) onto 2D graphene oxide, functionalized with epoxy and hydroxyl (HUGO) or carboxyl (TOGO) groups, and black phosphorus (BP) sheets. According to NP size, the deposition was conducted for various exposure times and several types of particle distribution. EDS confirmed the required chemical composition of all of the prepared materials. SEM showed the amount and distribution of the supported NPs, and TEM confirmed their size. Raman spectroscopy revealed a strong bonding between the NPs and the support sheets according to the type of 2D support. These results suggest that EBL is a promising method for the target-controlled deposition of metal NPs of targeted amount, size, and spatial distribution onto 2D materials, which enables evaluating the specific influence of the NP–support interaction on enhanced catalytic activity.

## Abstract 2

Direct electron beam lithography is studied for targeted platinum nanoparticle deposition onto 2D supports, which allows an evaluation of the unique influence of nanoparticle–support interactions on enhanced catalytic activity.

## Graphical abstract

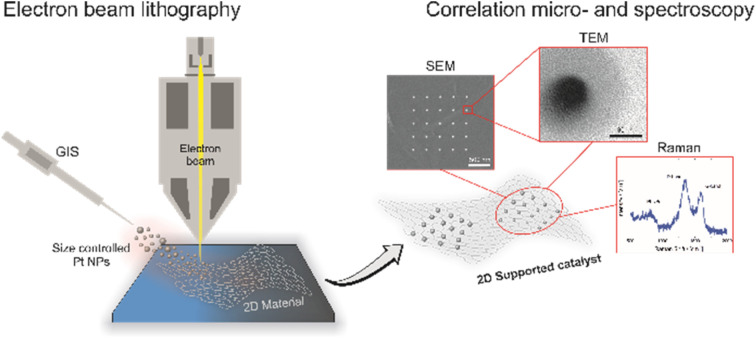

## 1. Introduction

Two-dimensional (2D) materials have attracted significant attention owing to their unique morphology, large surface area, and excellent optical and electrical properties. As a typical 2D material, graphene has been widely investigated for catalysis.^1–3^ Due to the perturbations in its perfect hexagonal structure, graphene can be modified to promote its catalytic properties. Recently, other 2D material families, such as black phosphorus,^4,5^ transition metal dichalcogenides (TMDs),^6^ and transition metal carbides and nitrides (MXenes),^7^ have been studied in the fields of photocatalysis, electrocatalysis, and thermocatalysis. Moreover, 2D materials are promising supports for metal nanoparticle-based catalysts.

Various studies have shown that 2D material-supported metal catalysts exhibit significantly improved catalytic performance. For instance, Wang *et al.*^8^ reported a 2D tungsten oxide (WO_3_) supported catalyst with enhanced activity for CO oxidation. The 2D WO_3_ nanosheets increased the stability of noble metals and improved the metal–support interaction, leading to a higher specific surface area and increased catalytic activity. Later, Lin *et al.*^4^ suggested a novel 2D material, black phosphorus (BP), as a support for the construction of a Ni_2_P-based hybrid catalyst. This catalyst exhibited high catalytic activity, conductivity, and good stability for the hydrogen evolution reaction, all of which were attributed to the strong interaction between the Ni_2_P NPs and BP. Thus, the enhanced catalytic performance of 2D material-supported metal catalysts appears to be due to the electronic properties of the 2D support and to the interaction between the metal nanoparticles (NPs) and support.^9–11^ However, the metal–support interaction is difficult to precisely qualify and quantify. To achieve this, the individual contribution of the NPs and support must be evaluated, and the only way of ensuring this request is to deposit identical patterns of supported NPs of defined size and spatial distribution on various 2D supports differing in physical, chemical, and electronic properties.

Several methods exist for the preparation of 2D material-supported metal nanoparticle catalysts, but each has drawbacks. Traditionally, impregnation has been the most common technique due to its simplicity. In this technique, a loading solution containing metal precursors is mixed with the support and then the solvent is evaporated. Slot *et al.*^12^ used this procedure to prepare platinum (Pt) NPs on MXenes as supports. Although the morphology of the prepared catalysts presented uniformly distributed metal particles, there were major differences in the particle size distribution. Another traditional method is co-precipitation, by which a solution of an active metal and a support are mixed, supersaturated, and grown as a combined solid precursor. This method was used by Ahmed *et al.*^13^ for the synthesis of two efficient catalysts based on CuAl- and CoAl-layered double hydroxides (LDH) supported on graphene oxide (GO). The synthesized catalysts demonstrated well-exfoliated GO sheets, completely covered by LDH particles. The morphological investigations of the LDH particles showed the lateral particle size of several hundred nanometers for CuAl-LDH and up to 8 μm for CoAL-LDH. Scanning electron microscopy (SEM) indicated clustering of the individual LDH particles into larger aggregates, while transmission electron microscopy (TEM) confirmed their hexagonal and platelet-like shape. At the same time, the packing of the individual LDH particles within the CoAl-LDH/GO sample appeared relatively random. Another method is hydrothermal synthesis, a solution reaction-based approach under high temperature and high-pressure water conditions. It involves crystal synthesis from substances that are insoluble at ordinary temperatures and pressures. Gao *et al.*^14^ used this approach to prepare noble metal NPs on a 2D Bi_2_WO_6_ nanosheet support. Although the morphology and structure of the material showed well-dispersed NPs on the surface of the Bi_2_WO_6_ nanosheets, the size distribution histogram of the metal NPs indicated a variation of particle size in the range from 3 to 8 nm and inhomogeneity. More recently, atomic layer deposition (ALD) has been developed as an effective method for the synthesis of supported catalysts. ALD is a gas-phase method based on a unique reaction mechanism, known as the self-limited surface saturation reaction. Mostly, two self-limited surface reactions occur and deposit a binary compound film, which leads to the deposition of a thin film with atomic level control.^15^ For this, gaseous reactants (precursors) are introduced into the reaction chamber to form the desired material *via* chemical surface reactions. This method was used by Lee *et al.*^16^ to deposit Pt NPs on 2D molybdenum disulfide (MoS_2_). The surface of the catalysts with Pt NPs exhibited an enhanced coverage with a quite homogeneous particle growth and the root-mean-square roughness of NPs was 1.15 ± 0.10, 0.95 ± 0.09 and 0.80 ± 0.12 nm at 10, 20, and 50 ALD cycles. Despite this, for the deposition of small and evenly distributed NPs, ALD requires activation of the MoS_2_ surface by plasma pretreatment. Mackus *et al.*^17^ combined ALD with electron beam-induced deposition (EBID) for the fabrication of Pt nanostructures. This technique included the preparation of a thin layer using EBID and the selective thickening of the prepared layer using ALD. The main advantage of this technique was the deposition of a high Pt content by treating the EBID layer to O_2_ before the ALD step. This improvement allowed the purification of the Pt NPs deposited by EBID and reduced the required electron dose to enhance the technique throughput. Although the throughput of the technique was improved, the overall preparation time consisted of two steps, and a pre-treated stage potentially reduces the competitiveness of this technique.

The above-described methods and studies show that controlling the particle density, size, and distribution of deposited NPs remains an unsolved challenge. However, electron beam lithography (EBL) could be ideal for the controlled fabrication of supported metal NPs on 2D sheet surface. The main advantage of EBL is that it is able to perform direct and targeted deposition on a desired 2D sheet of NPs independent of the local topography. This makes it possible to choose a sheet with specific characteristics, such as shape, chemical composition, and electronic properties, and leads to accessibility of deposited NPs for all characterization techniques. Moreover, EBL is a highly powerful tool that can prepare features with a precision of 10 nm^18–20^ at the desired spot of the material. These features are extremely useful for correlative spectro-microscopy, because they are big enough to be spatially distinguishable in determining a time-resolved microcatalytic activity using single-molecule fluorescence microscopy. Thus, EBL can be used to prepare an identical pattern of NPs on different 2D materials to investigate and evaluate the specific effect of the 2D support.

In this study, we present the first use of direct EBL incorporated in a dual-beam focused ion beam-scanning electron microscope (FIB-SEM) equipped with a gas injection system (GIS) to fabricate Pt NPs on 2D materials directly from a Pt organometallic precursor. We demonstrate the technique on the 2D materials: black phosphorus (BP) and graphene oxide, functionalized with epoxy and hydroxyl (HUGO) or carboxyl (TOGO) groups. The nanofabricated Pt NPs on 2D materials allow the specific interaction between the metal NPs and support to be comprehensively studied using model systems with different electronic properties of the support. The characterization of these systems allows us to evaluate the exclusive influence of the support on the intensity of the interaction between the active site on the prepared materials.

## 2. Experimental

### 2.1 Preparation of BP, HUGO, and TOGO sheets

BP was obtained by high-pressure conversion of red phosphorus with the following procedure:^21^ red phosphorus powder (10 g, 99.999%) was compressed into a pellet (20 mm in diameter), covered with a graphite foil, and placed in a uniaxial belt-type anvil cell. The conversion conditions were as follows: the pressure of 6 GPa at 600 °C, the heating and cooling rates per min with a dwell on 600 °C for 30 minutes. After this, the compressed BP pellet was mechanically separated from the graphite paper, grounded, and exfoliated using shear force milling (Ultra-Turax T18 milling device) in acetonitrile under an argon atmosphere. The exfoliation was carried out for 1 hour at a milling speed of 10 000 rpm.

HUGO was prepared by the commonly used Hummers' method.^22^ Graphite powder (5.0 g) and sodium nitrate (2.5 g) were added to cooled concentrated sulfuric acid (115 mL) at 0 °C and stirred for about 30 minutes to form a uniform mixture. The mixture was kept in an ice bath allowing the temperature not to rise above 20 °C. Then potassium permanganate (15 g) was added gradually to the above mixture with stirring and cooling. After removing the ice bath, the mixture was stirred and heated up to 35 °C for 30 minutes to thicken the reaction mixture into the paste. As soon as the thick paste was observed, deionized (DI) water (250 mL) was slowly added, and the obtained mixture was heated to 70 °C and was held at the constant temperature for 15 minutes. After that, the reaction was terminated by the addition of a large amount of DI water (1 L) and 3% of H_2_O_2_ solution, which was confirmed by a mixture color-changing from black to bright yellow. The obtained material was washed in DI water to remove the metal ions by repeated centrifugation. The washing procedure was repeated until a negative reaction on sulfate ions was reached. The dried GO was achieved by drying at 60 °C for 48 hours.

TOGO sheets were synthesized by Tour method.^23^ The solution of concentrated sulfuric and phosphoric acids (360 and 40 mL, respectively) was added to the mixture of graphite powder (3.0 g) and potassium permanganate (18.0 g). The reaction mixture was heated to 50 °C and stirred for 12 h. After cooling, the mixture was poured into a flask with ice (400 mL) and hydrogen peroxide (3 mL). The reaction mixture was filtered, and the filtrate obtained was centrifuged to decant the supernatant. Afterward, the remaining solid material was purified by multiple washing process in succession with deionized water, hydrochloric acid, and ethanol followed by repeated centrifugation. Finally, the obtained GO was vacuum-dried overnight at room temperature.

After material preparation, the exfoliation procedure was performed to obtain the individual graphene oxide sheets (HUGO and TOGO). For this, 400 mg of HUGO (TOGO) was dispersed in 100 mL of water by ultrasonication with a power of 400 W for 60 min.

### 2.2 Platinum deposition by EBL

**Fig. 1:**
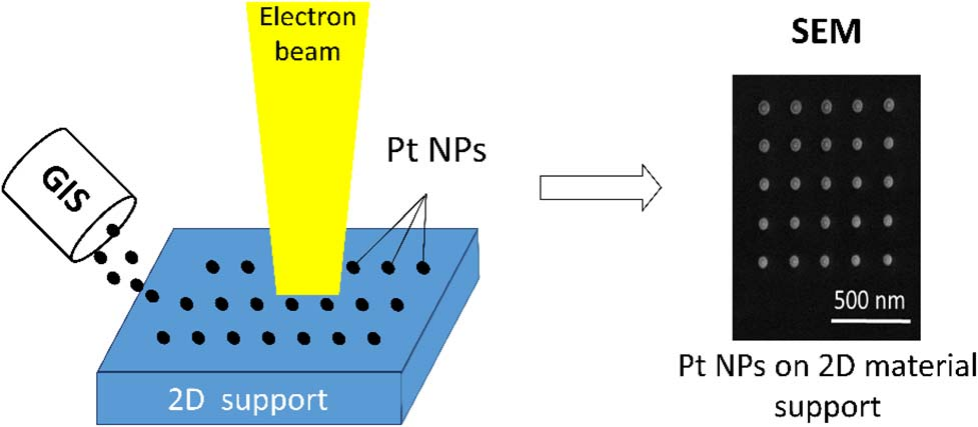
Illustration of Pt deposition onto 2D support by EBL.

For platinum deposition on a 2D support, direct EBL incorporated into a dual-beam FIB-SEM microscope (Lyra3 GMU Tescan, Czech Republic) equipped with a GIS (Ga^+^ LMIS) was used. Due to a GIS, the reacting molecules of a Pt organometallic precursor (OP) – trimethyl(methylcyclopentadienyl) platinum(iv) – were introduced as a gas in the working area and modified charged particle interactions with a 2D support surface. While the Pt OP gas was adsorbed on the surface of a 2D support due to van der Waals forces, the energy brought by the electron beam dissociated these molecules and formed the bonds between the Pt NPs and a 2D support. For deposition, the 2D supports were placed onto a TEM grid (Cu; 200 mesh; Formvar/carbon) or an ITO glass. Pt deposition was performed using precise working parameters for both the injected gas (injection pressure 1.5 × 10^−4^ Pa, nozzle temperature 90 °C) and the focused electron beam (beam current 170 pA, high voltage 30.0 kV, spot size 3.6 nm). In order to perform the deposition on a flat 2D nanosheet, the morphology of the 2D support was investigated as a preliminary step and the deposition was implemented on the specific flat spot of the chosen sheet. During deposition, the distance between a GIS and the 2D support surface was below 1 mm. According to Pt NP size, the deposition was conducted for various deposition (exposure) times ranging from 0.03 to 1.0 s (step 0.03 s) and several types of particle distributions (100–300 nm). For the purpose of confocal Raman microscopy with optical resolution limit of ∼200 nm, we decided to use the spatial distance between the NPs larger than this limit, although EBL itself allows unambiguously distinguish NPs in the distance of 20–30 nm (see ESI, Fig. S1†). It was necessary to deposit Pt NPs with a specific size, spatial distribution, and distance larger than the resolution of confocal microscopy (∼200 nm) to exclude the influence of the interaction between the active sites of the supports. For this reason, the deposition followed a regular square lattice pattern as the most beneficial structure-oriented form, which can be easily reproducible on various supports. A schematic illustration of the Pt deposition onto a 2D support by EBL is shown in Fig. 1.

### 2.3 Material characterizations

SEM was used to indicate the amount and spatial distribution of the supported NPs onto a 2D material. The measurements were performed on the Tescan Lyra3 dual-beam microscope with a field emission gun (FEG) electron source (Tescan, Czech Republic) using an accelerating voltage of 10 kV. Energy-dispersive X-ray spectroscopy (EDS) maps and spectra were acquired to confirm the required chemical composition of all materials using a silicon drift detector (SDD) (Oxford Instruments XMax 80, United Kingdom). The measurements were carried out under the next conditions: accelerating voltage – 20 kV; beam intensity – 12; energy range – 20 keV. With the AZtecEnergy software, the EDS maps were recalculated to quant maps. For raw materials, the samples were placed directly on carbon tape. For Pt NPs onto a 2D material, sample preparation was carried out by drop casting the suspension (3 mg mL^−1^ in isopropyl alcohol (IPA) on a TEM grid – Cu; 200 mesh; Formvar/carbon) followed by drying at room temperature.

X-ray diffraction (XRD) was performed to confirm the successful exfoliation and transformation of prepared layered materials into individual sheets. The measurements were conducted using a Bruker-Phaser 2nd Generation diffractometer equipped with Cu Kα radiation source (*λ*[Cu K-α_1_] = 1.54056 × 10^−1^ nm, *λ*[Cu K-α_2_] = 1.54443 × 10^−1^ nm) at room temperature in the range of 2*θ* from 4.99 to 90.019°.

Raman spectroscopy was used to provide structural characterization of sheets deposited with Pt NPs and to understand the bonding between the NPs and support. Raman measurements were carried out on an inVia Raman microscope (Renishaw, England) in backscattering geometry with a CCD detector. The green line of a diode-pumped solid-state (DPSS) laser (532 nm, 50 mW) was used as the excitation source. The Raman bands were collected with the applied power of 5 mW and 100× magnification objective in the wavelength range of 200–3000 cm^−1^ and grating of 2400 L mm^−1^ at room temperature. For instrument calibration, a peak position at 520 cm^−1^ was achieved on a silicon reference with a resolution less than 1 cm^−1^. The samples were suspended in IPA (3 mg mL^−1^), deposited on an ITO glass, and dried at room temperature. To characterize the specific 2D sheet with Pt NPs, the triangulation app was used. Raman maps were collected with a pixel size of 1.5 × 1.5 μm and processed using self-written code.

TEM and selected area electron diffraction (SAED) were performed for the characterization of pristine 2D materials and Pt NPs deposited onto a 2D material using an EFTEM Jeol 2200 FS microscope (Jeol, Japan) at an acceleration voltage of 200 keV. For the preparation of pristine material, the drop of the 2D material suspension (3 mg mL^−1^ in IPA) was deposited on a TEM grid and allowed to dry at room temperature (“drop casting”). For the characterization of Pt NPs deposited onto 2D materials, a regular square lattice pattern of NPs was deposited on all 2D materials by EBL using the GIS system incorporated in the dual-beam FIB-SEM microscope.

## 3. Results and discussion

### 3.1 Characterization of 2D support materials

The high-pressure conversion,^21^ Hummers^22^ and Tour^23^ methods were used to prepare the following 2D materials for the controlled deposition of Pt NPs: black phosphorus (BP); graphene oxide functionalized with epoxy and hydroxyl groups (HUGO); and graphene oxide functionalized with carboxyl groups (TOGO) (see Experimental 2.1). The prepared materials were characterized by a range of microscopic and spectroscopic techniques.

**Fig. 2:**
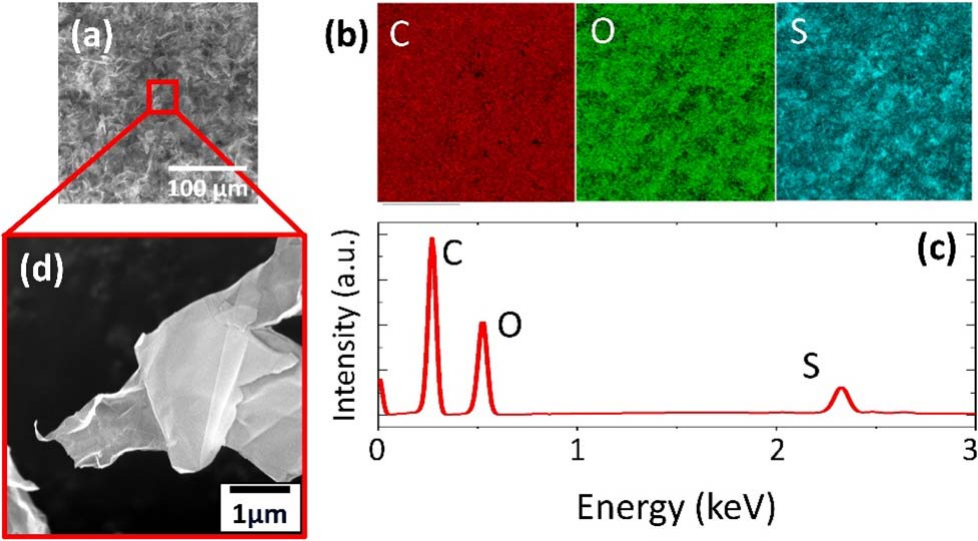
TOGO support material: (a) SEM image; (b) EDS mapping of elements obtained from SEM in (a): red – carbon, green – oxygen, blue – sulfur; (c) EDS spectrum; (d) magnified SEM image with detailed focus on single sheets.

**Fig. 3:**
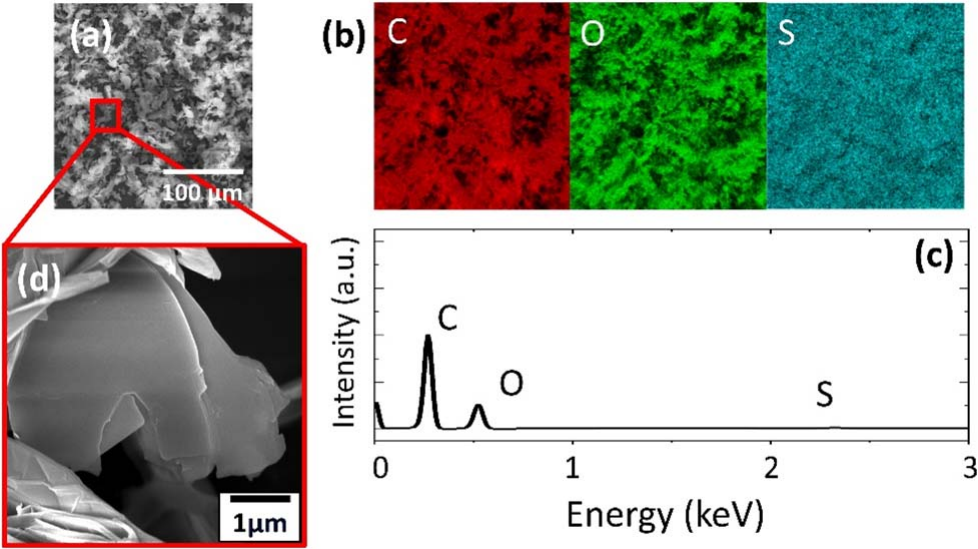
HUGO support material: (a) SEM image; (b) EDS mapping of elements obtained from SEM in (a): red – carbon, green – oxygen, blue – sulfur; (c) EDS spectrum; (d) magnified SEM image with detailed focus on single sheets.

**Fig. 4:**
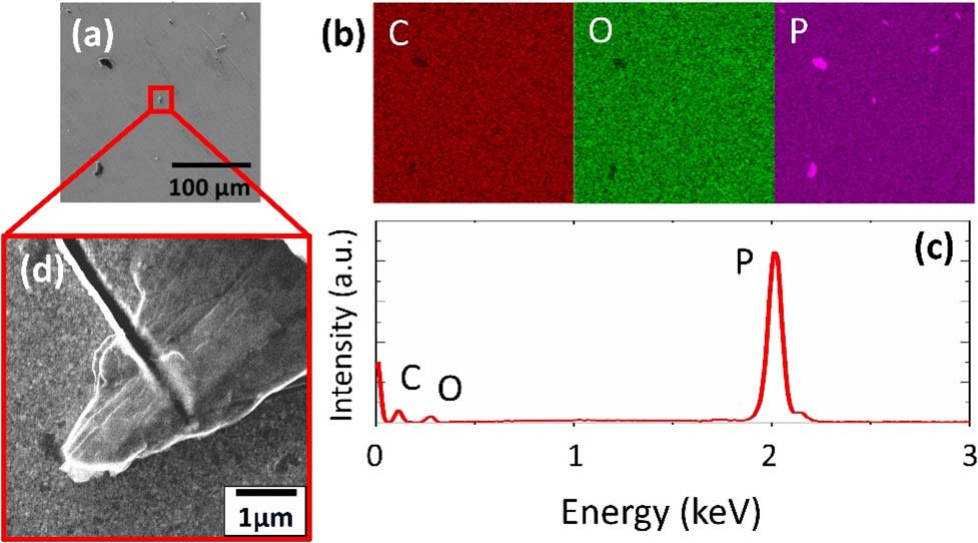
BP support material: (a) SEM image; (b) EDS mapping of elements obtained from SEM in (a): red – carbon, green – oxygen, purple – phosphorus; (c) EDS spectrum; (d) magnified SEM image with detailed focus on single sheets.

SEM revealed the morphology of the prepared 2D support materials, most of which had smooth flat surfaces that represented their layered structure (Fig. 2a, d, 3a, d, 4a and d). The lateral size of the 2D sheets predominantly ranged from 5 to 30 μm for both GO materials and from 10 to 50 μm for BP. Furthermore, the EDS maps (Fig. 2b, 3b and 4b) and spectra (Fig. 2c, 3c and 4c) indicated the presence of oxygen and carbon in the GO sheets, as well as the presence of phosphorus in the BP sheets. The presence of the contaminant sulfur was also observed in TOGO and HUGO (2.65 and 0.52 wt%, respectively), differing according to the preparation method (Table 1). In the case of the BP sheets, the presence of adventitious carbon and oxygen originates from the surface adsorption and surface oxidation of BP.^21^ Nevertheless, both SEM and EDS verified the formation of GO and BP 2D sheets.

**Table 1:** Elemental composition of 2D support materials

Element	Apparent concentration			*k* ratio			Weight [%]		
	TOGO	HUGO	BP	TOGO	HUGO	BP	TOGO	HUGO	BP
C	86.89	138.70	0.00	0.86893	1.38697	0.00002	56.22	65.84	5.76
O	76.08	68.76	0.00	0.25602	0.23139	0.00000	41.13	33.64	0.25
S	9.10	1.56	—	0.07839	0.01342	—	2.65	0.52	—
P	—	—	0.71	—	—	0.00399	—	—	93.99

**Fig. 5:**
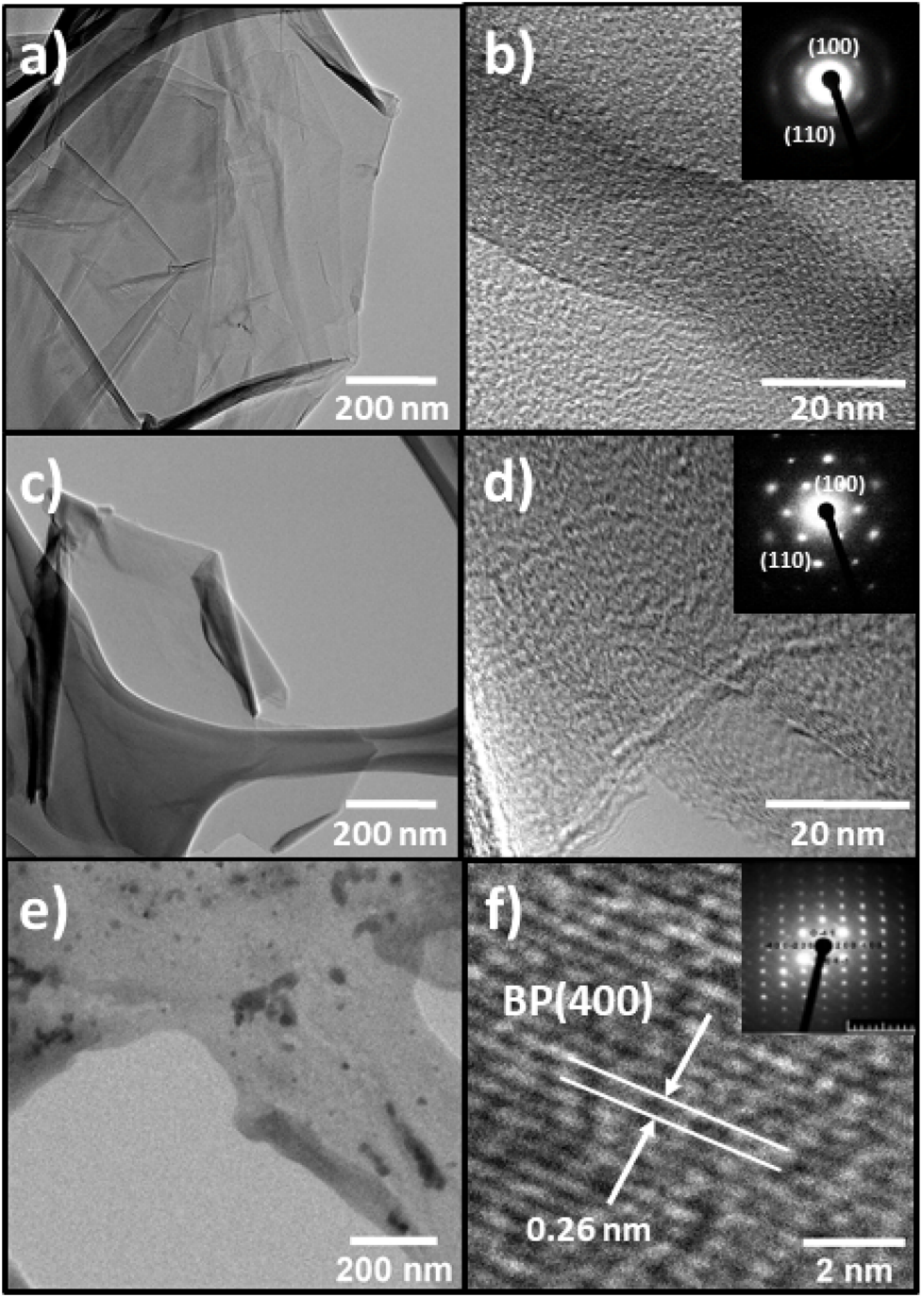
LR-TEM images: (a) TOGO sheet; (c) HUGO sheet; (e) BP sheet. HR-TEM images and electron diffraction: (b) TOGO sheet; (d) HUGO sheet; (f) BP sheet.

TEM confirmed the morphological properties of the 2D support materials and provided more detailed information regarding their crystal structure (Fig. 5). The low-resolution TEM (LR-TEM) of TOGO (Fig. 5a) showed twisted sheets entangled with each other, while the LR-TEM of HUGO and BP (Fig. 5c and e) exhibited few-layered nanosheets highly transparent for electrons.

Simultaneously, the high-resolution TEM (HR-TEM) of both GO types (Fig. 5b and d) revealed graphite lattices with ordered and disordered regions. This means that the graphene nanosheets were partly recovered to an ordered crystal structure. In addition, selected-area electron diffraction confirmed the 6-fold pattern (inset in Fig. 5b and d) of the diffraction spots for the GO materials, thereby indicating a crystalline order related to a hexagonal lattice. At the same time, the BP electron diffraction pattern disclosed a hexagonal lattice spacing of 0.26 nm from the BP plane [400] (Fig. 5f).

XRD provided valuable information regarding the crystal structure of the prepared materials. The XRD pattern of HUGO (Fig. S2†) demonstrated a strong and sharp peak at 13.15° corresponding to an interlayer spacing of 0.673 nm and indicating the presence of epoxy and hydroxyl functional groups in fully exfoliated graphene oxide sheets.

**Fig. 6:**
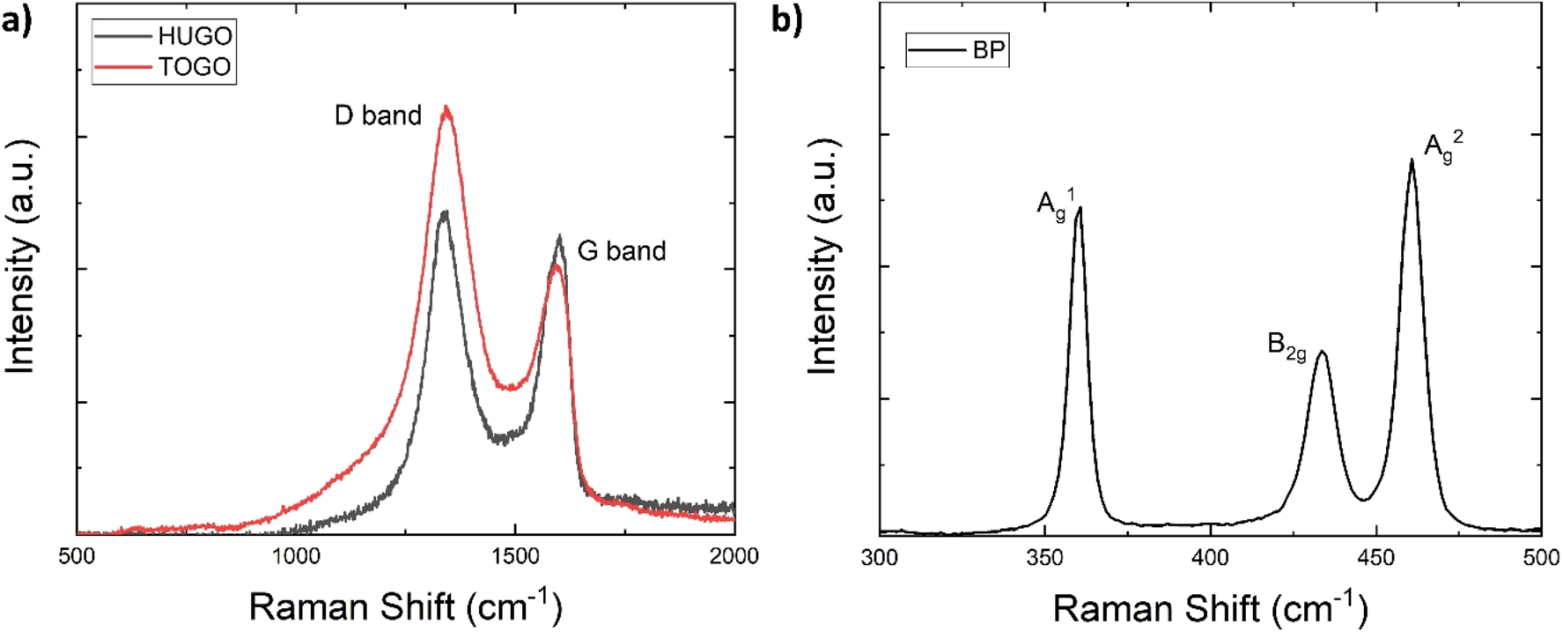
Raman spectra: (a) HUGO and TOGO; (b) BP.

Raman spectroscopy identified the chemical properties of the prepared 2D sheets by determining the main vibration modes of the GO and BP materials (Fig. 6). For HUGO and TOGO, the first peak, the G band, occurred at 1598 cm^−1^ and 1594 cm^−1^, respectively (Fig. 6a). Because this peak is usually observed at 1585 cm^−1^,^24^ this indicates a G band blue shift. The most plausible explanation for this blue shift is that an isolated C–C bond resonates at frequencies higher than the G band of graphite.^25^ The second peak, the D band, appeared at 1340 cm^−1^ and 1344 cm^−1^ for HUGO and TOGO, respectively (Fig. 6a); this is within the typical range of 1250 cm^−1^ to 1400 cm^−1^.^26^ Normalized intensity (*I*_D_/*I*_G_ ratio), used to quantify structural defects,^27^ was 1.35 and 2.03 for HUGO and TOGO, respectively (Tables 2 and 3). The higher *I*_D_/*I*_G_ ratio for TOGO is consistent with a broad and strong D peak indicating lattice distortions and a large number of sp^3^-like defects. This higher number of defects may be associated with the extensive oxidation of graphite and the destruction of its periodic structure during the functionalization of TOGO by carboxyl groups. In the case of BP, the three prominent peaks related to the A^1^_g_, B_2g_ and A^2^_g_ phonon modes occurred at 360, 433 and 460 cm^−1^, respectively (Fig. 6b). The presence of all main phonon modes in the Raman spectrum indicated the crystalline structure of the BP nanosheets after exfoliation.^28^

**Table 2:** Raman data for deposited Pt NPs on HUGO

Sample	Deposition time [s]	FWHM of G peak	FWHM of D peak	*I*_D_/*I*_G_	*L*_a_ [nm]
1	0	107.83	119.71	1.35	14.20
2	0.03	92.78	96.35	1.80	10.69
3	0.06	84.41	99.92	2.09	9.19
4	0.09	91.43	118.01	1.62	11.85
5	0.12	89.51	103.88	2.47	7.79
6	0.15	79.51	95.66	2.13	9.02
7	0.18	82.46	111.52	3.27	5.88
8	0.21	91.93	100.19	1.44	13.34
9	0.70	80.31	92.91	1.49	12.89

**Table 3:** Raman data for deposited Pt NPs on TOGO

Sample	Deposition time [s]	FWHM of G peak	FWHM of D peak	*I*_D_/*I*_G_	*L*_a_ [nm]
1	0	101.23	123.78	2.03	9.47
2	0.03	73.60	129.63	2.75	7.00
3	0.06	66.04	127.59	4.21	4.57
4	0.09	84.85	138.58	2.10	9.14
5	0.12	91.74	136.14	1.90	10.11
6	0.15	95.27	101.70	1.60	12.05
7	0.21	97.73	114.85	1.32	14.54
8	0.30	76.22	132.15	2.52	7.63
9	0.70	65.52	120.55	3.66	5.25
10	1.00	88.99	122.47	1.66	11.57

Overall, these techniques show that the prepared 2D materials not only exhibit excellent morphology in the form of few-layered nanosheets but also possess the required chemical composition and structure. Collectively, these attributes enable their use as supports for the targeted deposition of Pt NPs by EBL.

### 3.2 Characterization of Pt NPs deposited onto 2D support materials

#### 3.2.1 Morphological properties and chemical composition

**Fig. 7:**
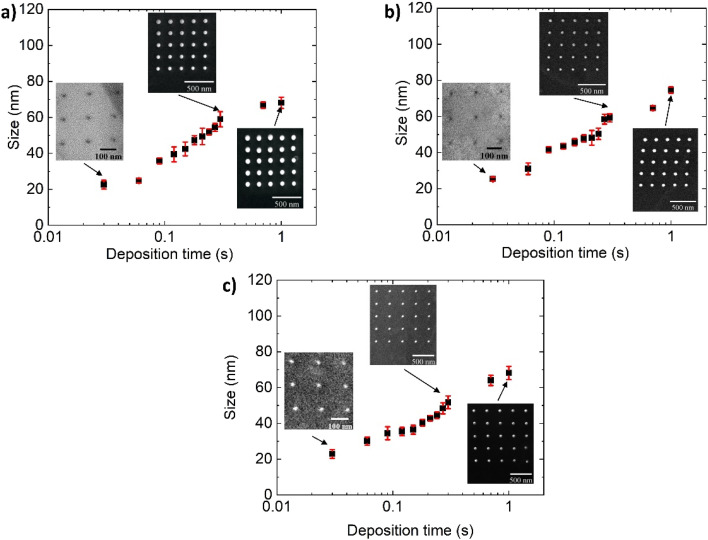
Dependence of Pt NP size from deposition time: (a) Pt/TOGO; (b) Pt/HUGO; (c) Pt/BP. Calculated values of confidence interval for each deposition time from statistical analysis of minimum 25 NPs.

SEM showed that the morphological structure of the Pt NPs directly deposited on the 2D supports by EBL exhibited a regular square lattice pattern in all cases (insets in Fig. 7a–c). Moreover, the insets exhibit that uniformly sized Pt NPs were deposited on targeted areas of the support in precise amounts and with the required spatial distribution. The size of Pt NPs increased as the exposure time enlarged (Fig. 7a–c), ranging from 23 ± 2, 23 ± 2 and 25 ± 1.2 nm to 68 ± 4, 68 ± 3 and 75 ± 2 nm for BP, TOGO and HUGO sheets, respectively. Interestingly, over the entire exposure time, the Pt NPs deposited on the HUGO sheets were always larger than those deposited on the BP and TOGO sheets, which were consistently around the same size. This phenomenon could be explained by the high chemical reactivity of BP and TOGO, which serves as a reductant in the deposition of Pt NPs.^29^ Consequently, the deposited NPs may be characterized by a higher Pt content due to the reduced number of impurities from the Pt organometallic precursor. Although SEM instrumental magnification was utilized to define the NP morphology, high-resolution TEM analysis was required to precisely estimate and confirm the distribution of the smaller NPs.

**Fig. 8:**
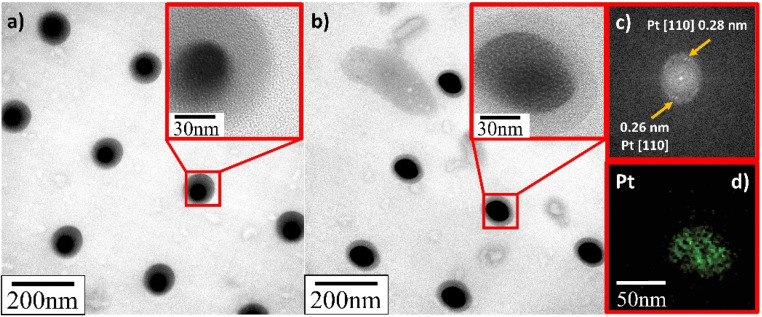
TEM and HR-TEM images of deposited Pt NPs on TOGO sheets for deposition time: (a) 0.3 s; (b) 1.0 s; (c) FFT pattern calculated from HR-TEM image in Fig. S3;† (d) EDS elemental map of platinum.

**Fig. 9:**
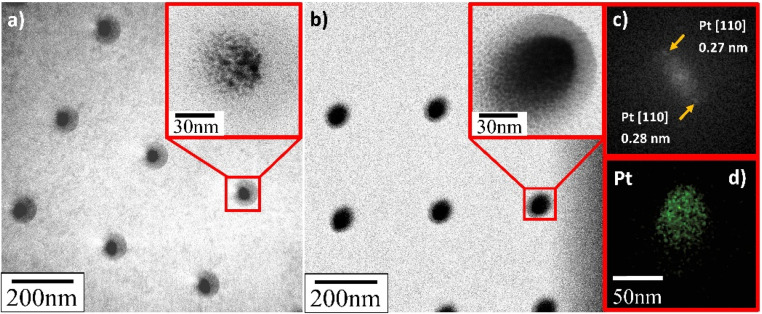
TEM and HR-TEM images of deposited Pt NPs on HUGO sheets for deposition time: (a) 0.3 s; (b) 1.0 s; (c) FFT pattern calculated from HR-TEM image in (b); (d) EDS elemental map of platinum.

**Fig. 10:**
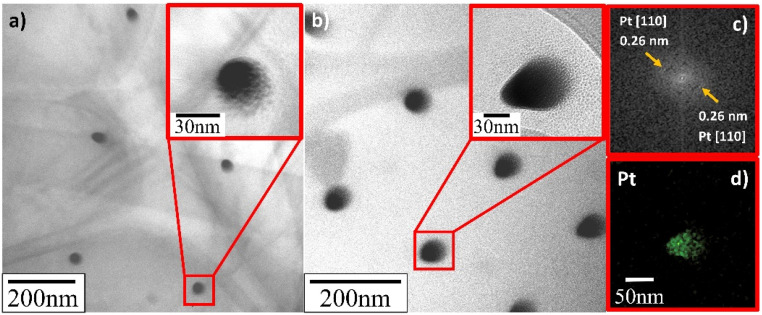
TEM and HR-TEM images of deposited Pt NPs on BP sheets for deposition time: (a) 0.3 s; (b) 1.0 s; (c) FFT pattern calculated from HR-TEM image in (b); (d) EDS elemental map platinum.

TEM verified that the supported Pt NPs were successfully deposited in an identical uniformly sized pattern. More importantly, and contrary to what SEM appeared to present, it revealed that the deposited Pt NPs were not individual particles but, in fact, the clusters of much smaller particles (insets in Fig. 8–10). The Pt/HUGO NPs exhibited the largest average size of 1.4 ± 0.3 nm, while the Pt/TOGO and Pt/BP NPs had similar size ranges of 1.3 ± 0.3 and 1.3 ± 0.2 nm, respectively (the pixel size of TEM images was 0.02 nm). Furthermore, the HR-TEM images shown in the insets in Fig. 8–10 and S2† demonstrate the formation of finely dispersed Pt NPs. Additionally, these HR-TEM images were used for FFT pattern calculations, in which diffraction spots attributed to the Pt [110] plane with interplanar distances of 0.28 nm (insets in Fig. 9b, c, 10b, c and S3†). As reported by Lin *et al.*,^4^ the high dispersion of metal NPs on 2D BP is probably due to the strong synergistic combination of NPs and BP. In the case of GO supports, several researchers have suggested that oxygenated functional groups act as anchor sites for Pt complexes supported on graphene oxide.^30^ Furthermore, these groups also play the main role in stabilizing the NP–support interface, which prevents NP sintering.^30^ Thus, the existence of smaller unsintered individual particles with good dispersion implies that more active sites can be displayed to increase catalytic activity.

Additionally, energy dispersive X-ray spectroscopy indicated the presence of Pt (Fig. 8d, 9d and 10d), as well as of O, C and P elements (Fig. S4†) in the Pt/GO and Pt/BP materials. However, an increase in C and O can also be observed in the area related to the deposited NPs. The most likely sources of increased C and O are the precursor molecules themselves or contamination from the SEM chamber and/or GIS needle.^31,32^ Despite this, the Pt profile recorded throughout the materials (Fig. S5†) clearly confirmed the formation of Pt in the prepared samples.

#### 3.2.2 Structural properties

**Fig. 11:**
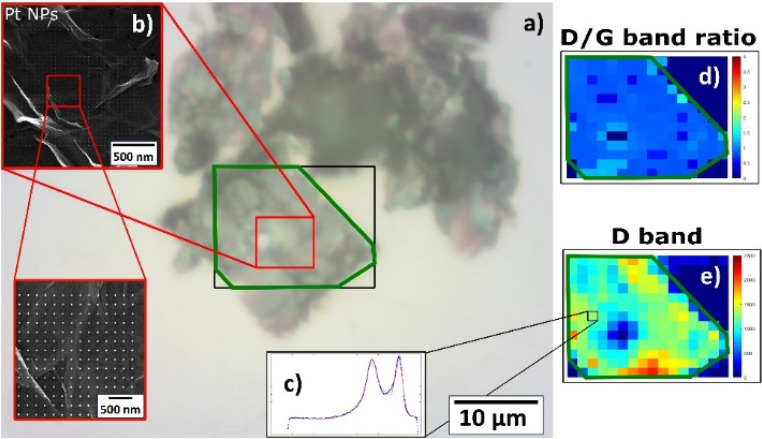
Raman characterization of deposited Pt NPs on TOGO sheet: (a) optical image of TOGO sheet with highlighted area of interest by green outline; (b) SEM image of deposited Pt NP precise spot studied *via* Raman mapping due to correlative approach; (c) Raman spectrum. Raman 2D contour plot of same spot from optical image highlighted by black rectangle: (d) D/G band ratio; (e) D band.

**Fig. 12:**
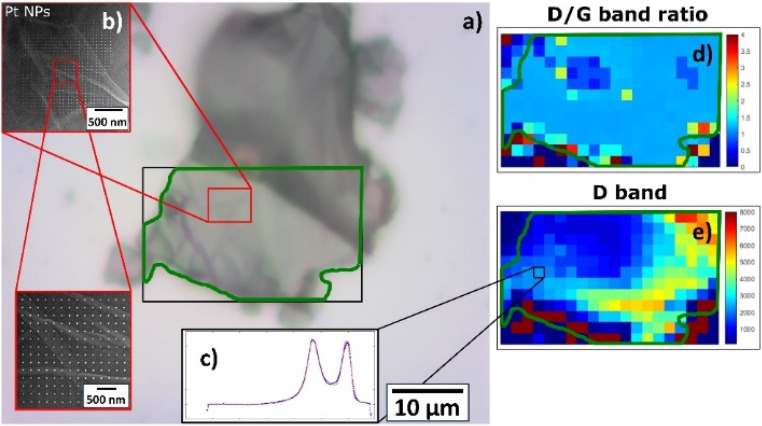
Raman characterization of deposited Pt NPs on HUGO sheet: (a) optical image of HUGO sheet with highlighted area of interest by green outline; (b) SEM image of deposited Pt NP precise spot studied *via* Raman mapping due to correlative approach; (c) Raman spectrum. Raman 2D contour plot of same spot from optical image highlighted by black rectangle: (d) D/G band ratio; (e) D band.

The structural properties of a catalyst influence the reactivity of active sites and, consequently, can enhance the catalytic performance. Raman spectroscopy revealed strong bonding between the Pt NPs and the support sheets, but different behavior according to the type of 2D support. For Pt NPs deposited on GO supports, regardless of the deposition time, the Raman spectra exhibited G and D vibration modes centered at 1585–1599 cm^−1^ and 1336–1348 cm^−1^, respectively (Fig. S6a and c†). More importantly, the spectrum of pristine HUGO had a lower intensity than its post-deposition spectra, while pristine TOGO had a higher intensity, with its strong and broad D peak indicating lattice distortions and high defect density. To quantify post-deposition defects, the full width at half maximum (FWHM) values of the D and G peaks were calculated by using a Gaussian fit for the Raman spectroscopy data and plotted as the *I*_D_/*I*_G_ ratio (Fig. S6b and d†). These ratios for Pt/HUGO decreased as the deposition time increased, while for Pt/TOGO the ratios first decreased and then increased. The *I*_D_/*I*_G_ ratios ranged from 1.44 and 1.32 to 3.27 and 2.75 for Pt/HUGO and Pt/TOGO, respectively (Tables 2 and 3), but a few highly defective points were detected corresponding to values up to 4.21. Furthermore, a 2D contour plot of the D/G band ratio (Fig. 11d and 12d) constructed from Raman maps exposed that changes in the ratio were related to the area covered by Pt NPs. In the figures, the optical images of Pt NPs deposited on TOGO (Fig. 11a) and HUGO (Fig. 12a) show the region of 2D sheets for measurements (green outline), while the SEM insets (Fig. 11b and 12b) demonstrate the precise spot of Pt NPs studied *via* Raman mapping owing to correlative microscopy approach. The 2D contour plots of the D/G band ratio and D band intensity (Fig. 11d, e, 12d and e) provided information about the uniform spatial distribution of the main peak intensities within the explored region, but the area covered by Pt NPs was characterized by the lower values, which is in good agreement with the decreased intensities of the D and G bands. The changes in the D/G band ratio can be attributed to the recovery of sp^2^-hybridized domains induced by Pt deposition.^33^

Thus, Pt NP deposition on the GO supports with the lowest exposure time led to a rise in the D-band intensity due to the introduction of structural defects. While the perfect structure of graphene prevailed over such defects, the intensity of the D band increased. A longer exposure time led to the amorphization of the crystalline structure and reduction of both Raman peaks.

**Fig. 13:**
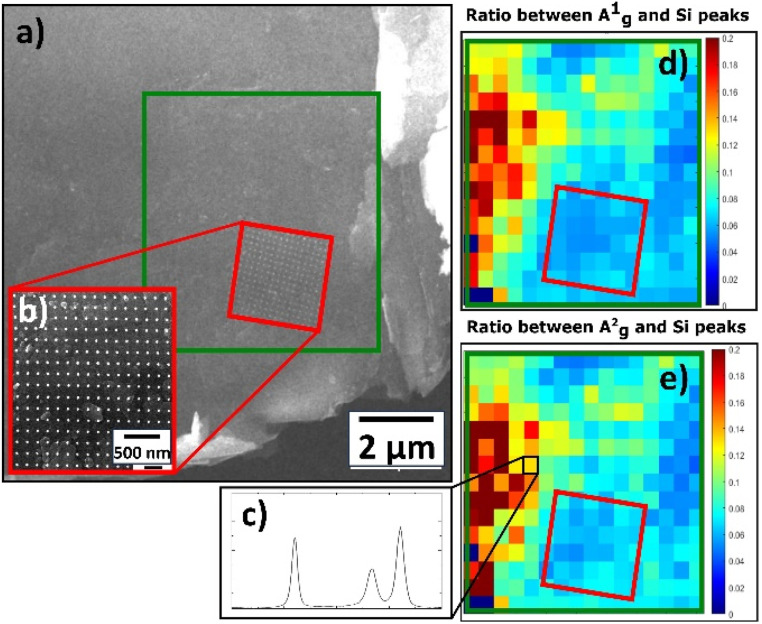
Raman characterization of deposited Pt NPs on BP: (a) SEM image of BP sheet with highlighted area of interest by green rectangle; (b) SEM image of Pt NP precise spot studied *via* Raman mapping due to correlative approach; (c) Raman spectrum. Raman 2D contour plot of same spot from SEM image highlighted by green rectangle: (d) A^1^_g_ band ratio; (e) A^2^_g_ band ratio.

The spectra of Pt NPs deposited on BP exhibited three main peaks (A^1^_g_, B_2g_, A^2^_g_) for all deposition times (Fig. S7†), but the general behavior of the spectra was the opposite of that observed for the Pt/GO materials; in other words, the intensity of the main peaks increased as the deposition time increased. The only exception to this was observed for A^1^_g_ at the deposition time of 0.27 s. However, the spectrum of pristine BP had the highest intensity of all peaks, which made the properties of pristine BP closer to the properties of pristine TOGO. Furthermore, the Pt/BP spectra exposed a significant Raman shift not only for the B_2g_ and A^2^_g_ in-plane modes, but also for the A^1^_g_ out-of-plane mode in the ranges of 459–465, 432–437, and 358–362 cm^−1^, respectively. According to the literature,^34^ Raman shifts of the B_2g_ and A^2^_g_ intraplane modes are dependent on the thickness of the sheet, but the A^1^_g_ interplane mode remains unchanged. In the case of Pt/BP, this A^1^_g_ phenomenon could be attributed to the presence of Pt NPs, similar to the anomalous behavior of E^1^_2g_ in MoS_2_ due to the presence of molybdenum atoms.^35,36^ Furthermore, the intensity ratios of A^1^_g_ and A^2^_g_ modes (Table 4) confirmed the decreased values within the area covered by Pt NPs (Fig. 13d and e), thereby confirming the influence of Pt deposition on the BP structure.

**Table 4:** Raman data for deposited Pt NPs on BP

Sample	Deposition time [s]	FWHM of A^1^_g_	FWHM of B_2g_	FWHM of A^2^_g_	A^1^_g_ band ratio	B_2g_ band ratio	A^2^_g_ band ratio
1	0	5.8361	8.1173	6.8066	0.0026	0.0036	0.0031
2	0.03	6.2332	10.0110	7.0276	0.0040	0.0064	0.0045
3	0.06	4.8002	5.6405	4.9276	0.0404	0.0475	0.0415
4	0.09	5.1594	6.2552	5.2171	0.0449	0.0544	0.0454
5	0.12	5.5340	7.8796	6.5187	0.0040	0.0057	0.0048
6	0.15	5.3442	6.6826	5.8866	0.0098	0.0123	0.0108
7	0.18	4.8088	5.6275	4.9844	0.0006	0.0007	0.0006
8	0.21	5.0124	6.0918	5.2188	0.0007	0.0009	0.0008
9	0.24	6.3771	9.2567	7.0803	0.0059	0.0085	0.0065
10	0.27	8.7104	11.9012	7.7900	0.0017	0.0023	0.0015
11	0.30	5.9336	8.3555	6.3300	0.0086	0.0121	0.0092
9	0.70	5.9944	8.7880	7.0131	0.0044	0.0064	0.0051
10	1.00	5.8266	8.4204	6.7112	0.0022	0.0032	0.0025

Simultaneously, Raman analysis was used to identify the in-plane crystallite size (*L*_a_) of the Pt/TOGO and Pt/HUGO materials, which was calculated by the following equation:^37^
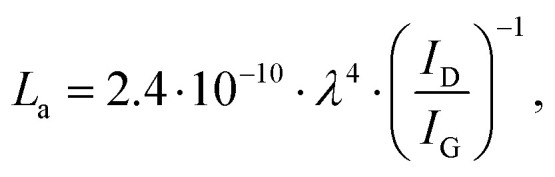
where *λ* is the wavelength of the excitation laser (532 nm) used in the Raman measurements.

The calculated crystallite size trend revealed that the average domain sizes of Pt/TOGO and Pt/HUGO do not depend on Pt deposition. For the former, the average crystallite size first decreased and then increased, while for the latter, the size decreased. For pristine TOGO and HUGO, *L*_a_ was 9.47 and 14.20, respectively. For the Pt/TOGO and Pt/HUGO NPs, the *L*_a_ distribution ranged from 4.57 to 14.54 (Table 3) and from 5.88 to 13.34 (Table 2), respectively. Such variations in size values can be attributed to a susceptibility of TOGO and HUGO to a high-temperature exfoliation and affected by the number of GO layers.^38^

Overall, Raman analysis revealed that Pt deposition caused structural changes in graphene oxide and black phosphorus, which were proven not only by the decrease in *I*_D_/*I*_G_ ratio values and the increase in the A^1^_g_ and A^2^_g_ intensity ratios, but also by a substantial Raman shift for all modes in the Pt/BP spectra. These structural changes were driven by the dependence of Pt NP size on the deposition time, thereby enabling the controlled deposition of identical NP patterns, with fixed density and spatial distribution, using electron beam lithography. Thus, varying the deposition time and 2D support makes it possible to control the structural properties of a prepared material to enhance its catalytic activity.

## 4. Conclusions

To our best knowledge, we are the first to report the utilization of direct electron beam lithography, incorporated in a dual-beam FIB-SEM microscope equipped with a gas injection system, to fabricate Pt NPs on 2D support materials with the controllable density, size, and distribution. This method allowed us to prepare an identical pattern of Pt NPs on three 2D supports (BP, HUGO, and TOGO) to evaluate the specific influence of the NP–support interaction on enhanced catalytic activity. SEM confirmed a regular square lattice pattern in all cases and showed that Pt NPs increased as the deposition time enlarged. Simultaneously, TEM revealed that Pt NPs were the clusters of smaller individual particles. The Pt/HUGO NPs had the largest average size of 1.4 ± 0.3 nm, while the Pt/TOGO and Pt/BP NPs had a similar size of 1.3 ± 0.3 and 1.3 ± 0.2 nm, respectively. Furthermore, the HR-TEM images demonstrated the formation of finely dispersed Pt NPs on the surface of 2D supports, which can be assumed to the strong synergistic effect between NPs and 2D material due to a high chemical reactivity of the latter. Moreover, oxygenated functional groups on the GO surface played the major role in stabilizing the NP–support interface, which led to the prevention of NP sintering and indicated that more active sites can be disclosed to enhance catalytic activity. Raman measurements exposed that Pt deposition changed the nature of black phosphorus and graphene oxide based on the dependence of Pt NP size on the deposition time. These changes were confirmed by the intensity ratios of increase in the A^1^_g_ and A^2^_g_ and the decrease in *I*_D_/*I*_G_ ratio values. Therefore, varying only the 2D support materials with different electronic properties and the deposition time provides an opportunity to control the structural properties of a prepared material to increase its catalytic activity. It also seems that the optimized parameters of the NP microstructure, such as particle size and spatial distribution, could be applicable to other preparation methods. Thus, the obtained Pt NPs on 2D support material enable a particular investigation of the specific interaction between the metal NPs and the support.

## Data availability

Data for this article, including SEM and TEM images, EDS maps, Raman spectra and maps are available at Zenodo repository at https://doi.org/10.5281/zenodo.14186160.

## Author contributions

ID: writing – original draft, visualization, methodology, investigation, formal analysis, data curation. LK: visualization, investigation, formal analysis. KK: investigation, data curation. TH: methodology, formal analysis. MP: supervision, investigation, formal analysis, data curation. JS: methodology, formal analysis, data curation. ZS: methodology, investigation, funding acquisition. MV: writing – review & editing, writing – original draft, visualization, supervision, methodology, formal analysis, data curation, funding acquisition.

## Conflicts of interest

There are no conflicts to declare.

## Supplementary Material

NA-OLF-D5NA00036J-s001
