# Supplementary material for: Direct chemical lithography writing on 2D materials by electron beam induced chemical reactions
Source: Nanoscale Adv. 2025 Feb 10;7(7):2021–31. doi: 10.1039/d5na00036j (PMC11833312; doi:10.1039/d5na00036j)
Supplement: NA-007-D5NA00036J-s001 [file NA-007-D5NA00036J-s001.pdf]

Supplementary Information (SI) for Nanoscale Advances.
This journal is © The Royal Society of Chemistry 2025

Direct Chemical Lithography Writing on 2D Materials by Electron Beam Induced Chemical Reactions

Iryna Danylo,[1] Lukáš Koláčný,[1] Kristína Kissíková,[1] Tomáš Hartman,[2] Alena Michalcová,[3] Martina Pitínová,[1] Jiří Šturala,[2] Zdeněk Sofer*[2] and Martin Veselý*[1]

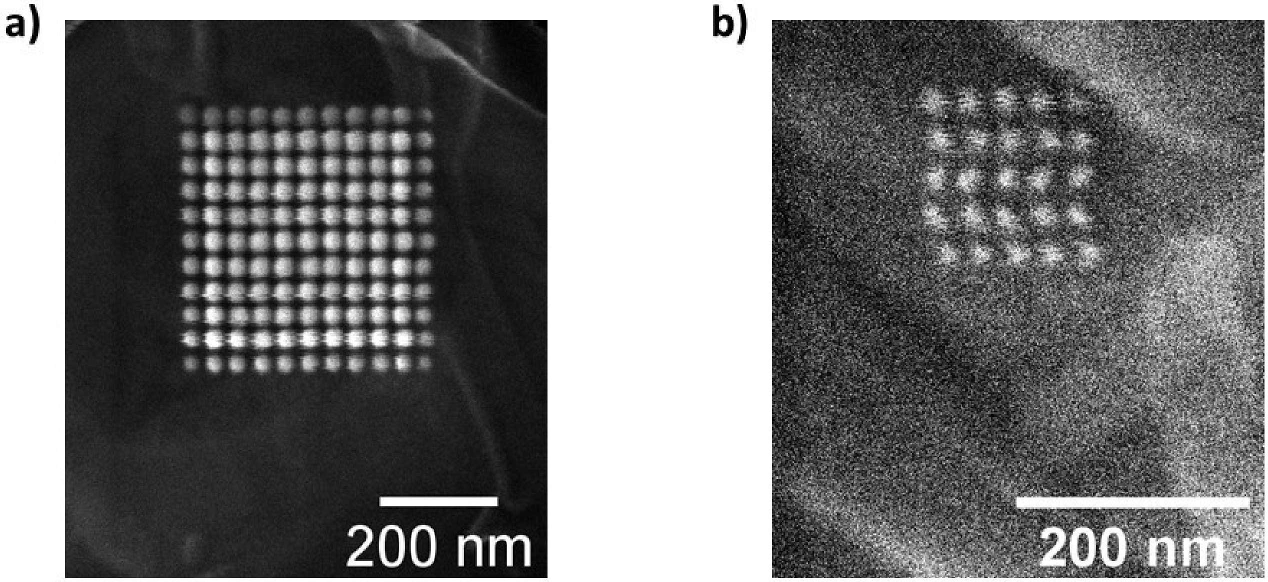

Fig. S1 SEM images of deposited Pt NPs on TOGO sheet by EBL. Deposition time: (a) 0.03 s; (b) 0.01 s. Distance between NPs: (a) 40 nm; (b) 30 nm

SEM images clearly confirmed the possibility for EBL to unambiguously distinguish Pt NPs with spatial distributions of up to 30 nm. According to Fig. S1a, the regular square lattice pattern of individual NPs can be nicely seen for the deposition time of 0.03 s and spatial distance between NPs of 40 nm. Furthermore, decreasing of deposition time up to 0.01 s allowed to deposit NPs with spatial distribution of 30 nm (Fig. S1b). However, the contrast between NPs and support became almost negligible for SEM to distinguish NPs, while EBL itself is able to deposit NPs at distance up to 10 nm.

[1] Department of Organic Technology, University of Chemistry and Technology, Prague, Czech Republic
[2] Department of Inorganic Chemistry, University of Chemistry and Technology, Prague, Czech Republic
[3] Department of Metals and Corrosion Engineering, University of Chemistry and Technology, Prague, Czech Republic

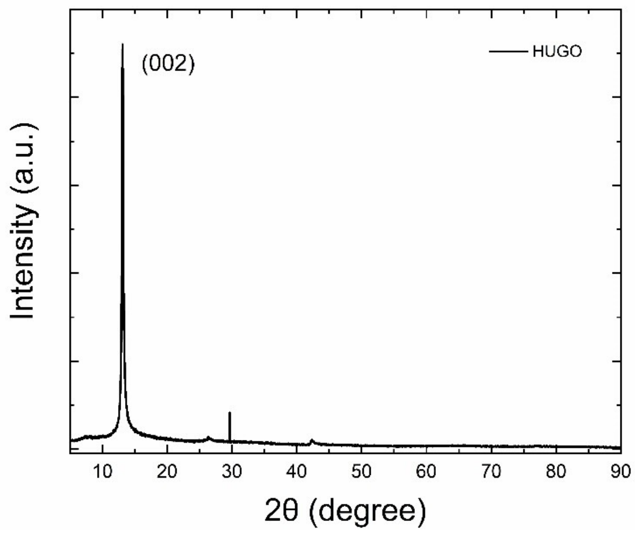

Fig. **S2** XRD spectrum of HUGO support

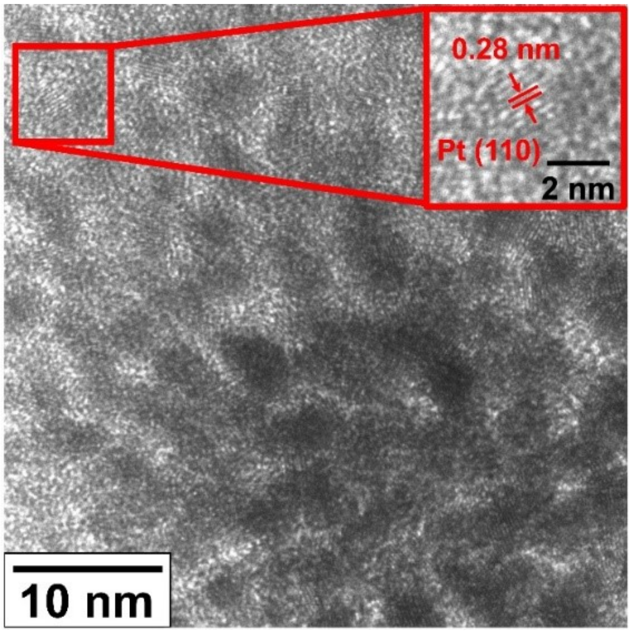

Fig. **S3** HR-TEM image of Pt/TOGO NPs used for FFT pattern calculation with focus on Pt lattice structure corresponding to Pt [110]

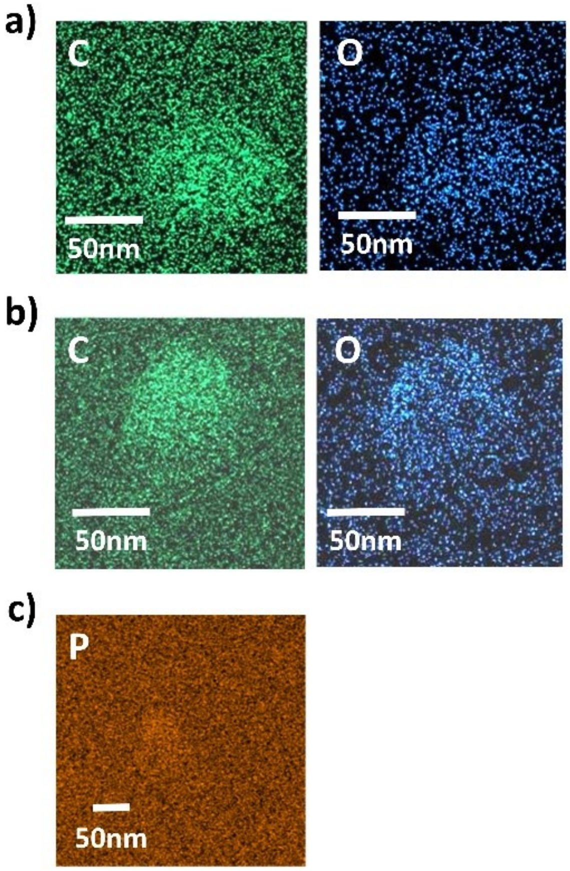

Fig. **S4** EDS elemental mapping images of C, O and P from deposited Pt NPs: (a) TOGO sheet; (b) HUGO sheet; (c) BP sheet

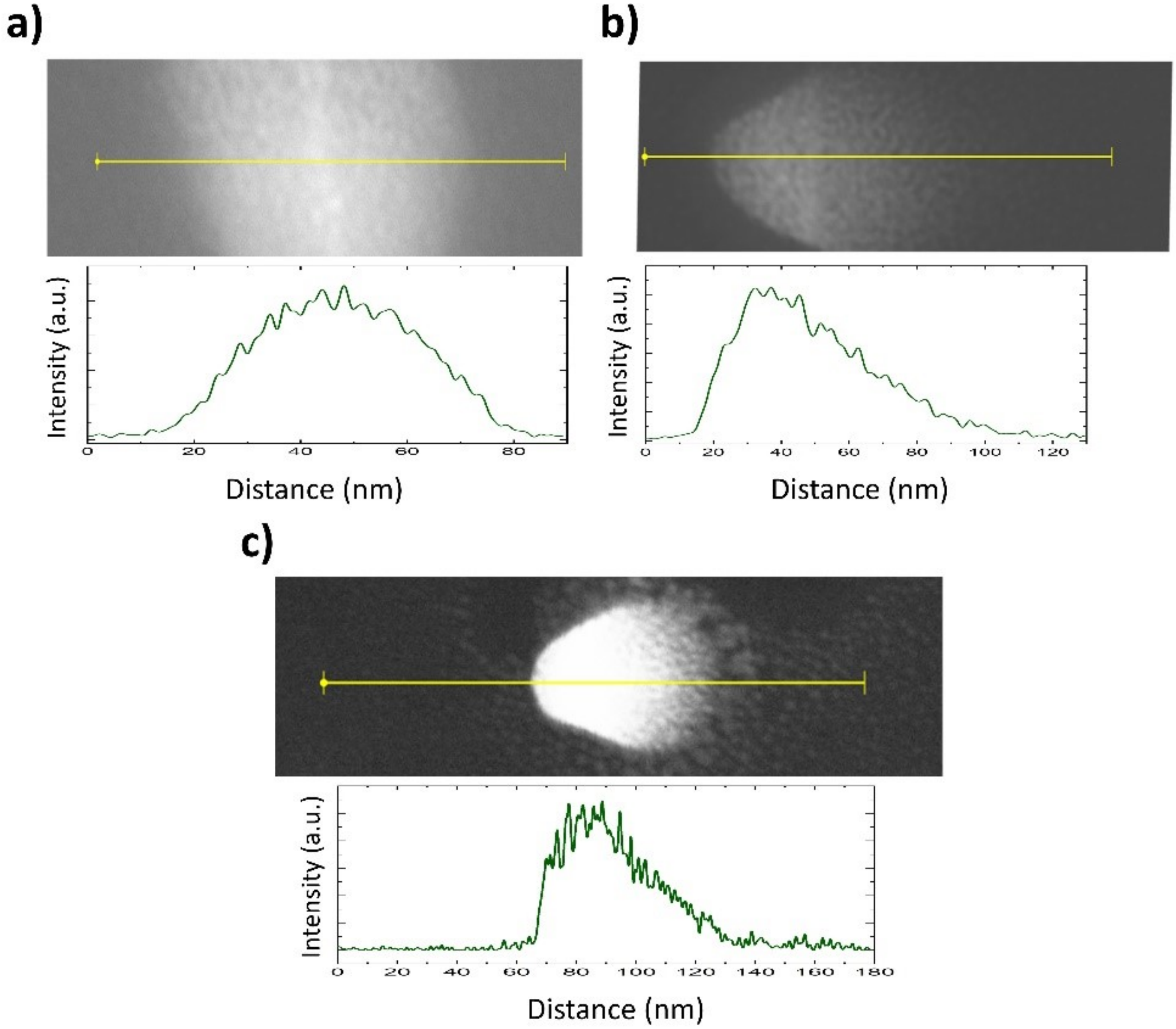

Fig. **S5** Profile of deposited Pt NPs: (a) TOGO; (b) HUGO; (c) BP

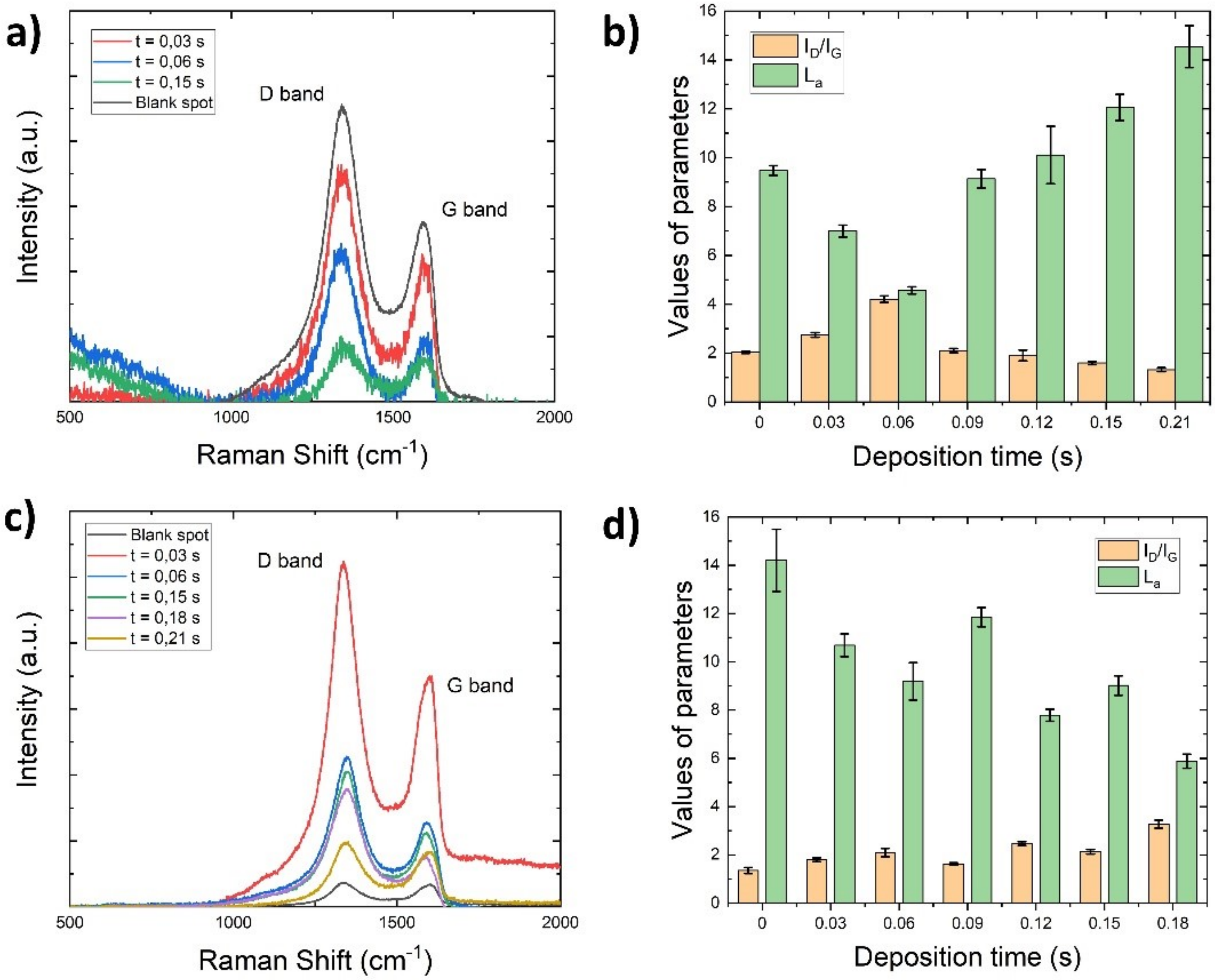

Fig. **S6** Raman spectra of deposited Pt NPs: (a) TOGO; (c) HUGO. Computed values of I_D/I_G ratio intensity and crystallite size L_a for Raman spectra of deposited Pt NPs: (b) TOGO; (d) HUGO

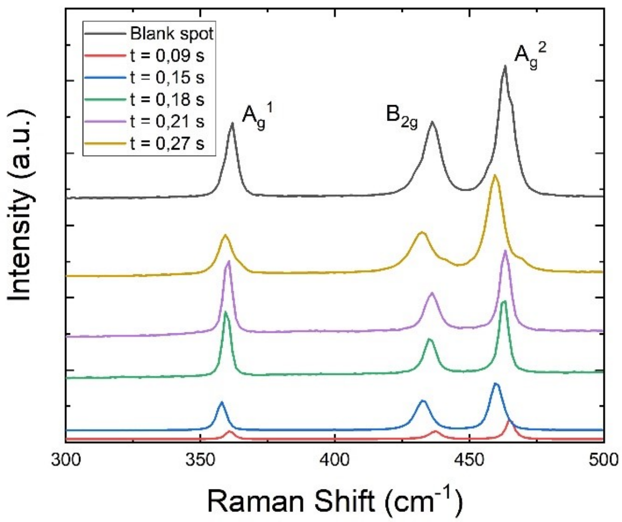

Fig. **S7** Raman spectra of deposited Pt NPs on BP
